# Effects of Mulligan Mobilization and Low-Level Laser Therapy on Physical Disability, Pain, and Range of Motion in Patients with Chronic Low Back Pain: A Pilot Randomized Controlled Trial

**DOI:** 10.3390/healthcare8030237

**Published:** 2020-07-29

**Authors:** U-Hyeok Seo, Jung-Hee Kim, Byoung-Hee Lee

**Affiliations:** 1 Graduate School of Physical Therapy, Sahmyook University, Seoul 01795, Korea; Seowuhyeok@naver.com; 2 Department of Physical Therapy, Andong Science College, Andong 36616, Korea; mirrorneuron98@gmail.com; 3 Department of Physical Therapy, Sahmyook University, Seoul 01795, Korea

**Keywords:** manipulation therapy, low-level laser therapy, low back pain

## Abstract

This study aimed to determine the combined treatment effects of Mulligan sustained natural apophyseal glides (SNAGs) and low-level laser therapy (LLLT) on function, pain, and range of motion (ROM) in patients with chronic low back pain. A total of 49 adults participated in this study and were randomly divided into three groups (SNAGs with LLLT group, SNAGs group, and control group). The participants in the SNAGs with LLLT group received SNAGs for 10 min, LLLT for 10 min, and electrotherapy for 10 min. The SNAGs group received SNAGs for 10 min and electrotherapy for 20 min. The control group received electrotherapy for 30 min. All participants received the assigned treatment for 30 min a day, 3 times a week, for 4 weeks. We used the visual analogue scale (VAS) to measure pain, the modified-modified Schober test (MMST) to measure ROM, and the Roland Morris disability questionnaire (RMDQ) to measure physical disability. Compared to the pre-intervention values, the VAS and MMST scores significantly increased after the intervention in the SNAGs with LLLT group (*p* = 0.000) and the SNAGs group (*p* = 0.000). The RMDQ score significantly improved in the SNAGs with LLLT (*p* = 0.000), SNAGs (*p* = 0.000) and control (*p* = 0.025) group after the intervention. The inter-group differences were greater for the SNAGs with LLLT and SNAGs groups than for the control group (*p* = 0.001), and the difference was greater for the SNAGs with LLLT than for the SNAGs (*p* = 0.001) with respect to the VAS, MMST, and RMDQ scores. These results indicate that significant improvement in pain, function, and ROM may be achieved by a combination of SNAGs and LLLT to treat chronic low back pain.

## 1. Introduction

Low back pain is a common condition noted in humans. Statistically, 70–80% of the population experiences low back pain in their lifetime [1,2]. The prevalence of low back pain is consistently increasing. It is an important cause of absenteeism from work and disability, thus affecting an individual’s socioeconomic status [3]. In 10–20% of the cases, back pain develops into chronic low back pain, which is associated with pain and disability persisting for more than 12 weeks [4]. Specific low back pain is defined as low back pain with a specific pathoanatomic origin, such as a tumor or fracture, and appropriate treatment, such as medication or surgery, is required in such cases. However, in 90% of the cases of low back pain, a precise specific origin of the pain cannot be identified, and such pain defined as nonspecific low back pain [3]. Chronic low back pain is defined as lumbosacral pain with or without pain referred to other body parts [5]. Additionally, chronic low back pain may lead to abnormal movements of the spine [5]. Back pain develops owing to degeneration of the nucleus pulposus, musculoskeletal sprains, abnormal location of the spine, or movement disabilities [5]. Low back pain is generally noted in the herniated nucleus pulposus (39%), facet joints (15%), and sacroiliac joints (13%). Each structure is connected to nociceptive, mechanical, and chemical receptors that cause low back pain [6,7].

Spinal injection therapy, surgery, and opioid administration are the treatment strategies used most frequently in clinical practice for patients with chronic low back pain [8]. However, these strategies do not always yield significant clinical outcomes. It is difficult to expect an effect on other complications of opiate analgesics that can induce mutations in the brain, spinal cord, and peripheral nervous system [9]. Mulligan mobilization with movement (MWM) is widely used during physical therapy and orthopedic manual therapy and can be applied to the peripheral and spinal joints [9]. When MWM is applied to the spinal joints, it is called sustained natural apophyseal glides (SNAGs) [10]. SNAGs is a mobilization technique that improves joint mobility through the application of passive gliding to the lumbar spine while the subject simultaneously performs active movement [11]. Hidalgo reported significant differences in the visual analogue scale (VAS) score and range of motion (ROM) of the joint and kinesiophobia between the SNAGs and placebo groups, comprising 32 patients with chronic low back pain, after two weeks of treatment [3]. Mulligan assumed that limited facet joint gliding in flexion may induce pain owing to the deformation of the nucleus pulposus. Therefore, normalizing facet joint movement may aid in the resolution of pain [10].

To reduce pain, ultrasound, low-level laser therapy (LLLT), heat and cold treatment, interferential current therapy (ICT), and transcutaneous electrical nerve stimulation are performed by physical therapists [12]. Laser therapy (LLLT and high-level laser therapy (HLLT)) is widely used for treating musculoskeletal diseases. The wavelengths used in both HLLT and LLLT are approximately 600–1000 nm. The output of HLLT is 5–10 W [13]. HLLT induces a thermal effect and is commonly used in surgery. The output of LLLT is limited to 500 mW. The main effects of LLLT are pain reduction and functional improvement. There is evidence indicating that LLLT manipulates the inflammatory, proliferative, and remodeling phases of the healing process [14,15]. In addition, the analgesic effect of LLLT is mediated through the suppression of the synaptic activity in second order neurons; hence, the pain processing area of the cortex is not activated [15].

Previous studies have investigated the efficacy of combining other mobilization or manipulation techniques with LLLT for treating low back pain in some parts of the body, such as the neck and wrists [16,17]. However, a study investigating the combined treatment effects of Mulligan mobilization with LLLT on patients with chronic low back pain has not been conducted to date. Therefore, this study was conducted to determine the combined treatment effects of Mulligan mobilization and LLLT on pain, ROM, and function in patients with chronic low back pain.

## 2. Materials and Methods

### 2.1. Methods

The subjects of this study were 49 patients with chronic low back pain who were undergoing physical therapy at Newstart Hospital in Seoul, Korea. The inclusion criteria were as follows: a 12 week history of chronic low back pain, aged 18–55 years, pain rating of 3–8 cm on the VAS, and 20–80% functional disability, as assessed on the Roland Morris Disability Questionnaire (RMDQ) [18,19]. In contrast, the exclusion criteria were as follows: pregnancy, obesity, contraindication for physical and manual therapy, history of fractures, tumors, lumbar stenosis, neuropathy, radiculopathy, rheumatoid arthritis, lumbar spine surgery, and long-term use of steroids. These factors were considered as exclusion criteria because these factors were associated with the exacerbation of symptoms or serious damage [20]. The study protocol was approved by the Sahmyook University Institutional Review Board (2-7001793-AB-N-012019002HR), and it was registered (KCT0005094) on Clinical Research Information Service (CRIS) in the Republic of Korea. The objective and the procedures performed in the study were fully understood by the subjects, and all subjects provided informed consent for inclusion in the study. This study was based on the ethical principles of the Declaration of Helsinki.

### 2.2. Sample Size

The sample size was estimated based on the primary endpoint, which was defined as the immediate effect of the intervention on the VAS, modified-modified Schober test (MMST), and RMDQ scores. The overall effect size index for all the outcome measures and power of the study was 0.30. To minimize type II errors (power of 95%), a sample size of 16 subjects was required for each group. The sample size estimates were calculated using G*power Version 3.1.9.7 (Franz Faul, University Kiel, Kiel, Germany, 2020).

### 2.3. Experimental Methods

**Figure 1:**
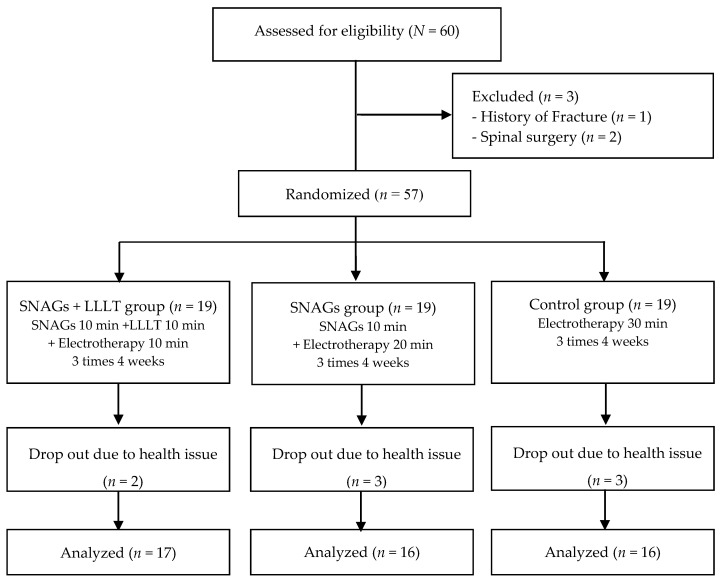
Schematic of the study design. SNAGs = sustained natural apophyseal glides; LLLT = low-level laser therapy.

A total of 60 qualified subjects were recruited, excluding three subjects who met the exclusion criteria. A total of 57 patients participated in the study, and eight dropped out during the course of the study. Demographic data such as age, weight, height, and body mass index, were collected before the pretest. All subjects picked a piece of paper with number one, two and three written on it from a box containing 57 pieces of paper. Subjects were randomly divided into three groups for SNAGs with LLLT group, SNAGs group, and control group. The SNAGs with LLLT group received for 10 min, LLLT for 10 min, and electrotherapy for 10 min. The SNAGs group received SNAGs for 10 min and electrotherapy for 20 min. The control group received electrotherapy for 30 min (Figure 1).

### 2.4. Procedures for Mulligan Mobilization (Sustained Natural Apophyseal Glides)

First, the symptomatic spinal level was determined by performing standardized clinical examinations. Subjects were asked to perform active trunk flexion and extension to identify which motion induced greater pain and the most painful vertebral level by applying passive accessory intervertebral movement [20]. Second, subjects were seated on a height-adjustable table while both feet were placed on a footrest slightly plantar flexed. The Mulligan belt was applied around the anterior superior iliac spine (ASIS) of the subjects and the hip joint of the therapist [21]. SNAGs were applied on the transverse process of the symptomatic spinal level of the subject with hypothenar. Direction of mobilization was parallel to the facet joint plane (cranial direction). Subjects were asked to lean forward while the therapist applied mobilization force simultaneously. After reaching the end range of flexion, subjects were instructed to return to the starting position. A cranially directed mobilization force was maintained while the subjects were returning to the starting position [3]. To minimize the therapeutic differences among therapists, SNAGs were performed by physical therapists with more than five years of clinical experience and who had completed the Orthopedic Manual Physical Therapy course in Korea.

### 2.5. Procedures for Low-Level Laser Therapy

In this study, the Therap HLA-200 (Hanil TM, Daegu, Korea) was used for administration of LLLT. It was administered to the subjects using a handheld probe; a laser beam at a dose of 27 J/cm^2^ was focused on the eight most painful points in the paravertebral areas (L2 to S3), including the apophyseal capsules, dorsolumbar fascia, and interspinous ligaments for over 20 min for each session. The pain points of the participants were identified via palpation and marked prior to administration of laser treatment. Each point was irradiated for 90 s [22]. Both the subject and examiner wore protective glasses.

### 2.6. Procedures for Conventional Physical Therapy

Electrotherapy was performed in the SNAGs with LLLT group, SNAGs group, and control group. Hot pack treatment was administered for 10 min and ICT (IFC ALPHA1/Japan) was administered for 20 min to the most painful areas. For patients with bilateral low back pain, the “painful area” placement technique was performed, wherein electrodes were placed parallel to the vertebral column covering the margin of the painful area. For patients with unilateral low back pain, the spinal nerve root electrode placement technique was performed. The proximal electrode was placed 2 cm lateral to the intervertebral foramen, and the distal electrode was placed another 2 cm away lateral to the proximal electrode. Both the electrodes were placed parallel to the vertebral column level [23].

### 2.7. Outcome Measures

We used the VAS to identify the effects of treatment on chronic low back pain. Horizontal non-numeric VAS was used. A score less than 10 mm was considered indicative of “no pain”, and of 100 mm was considered indicative of “the most extreme pain that can be ever experienced”. For chronic low back pain, the minimal clinically important difference (MCID) was approximately 18–20 mm [24]. The intraclass correlation coefficient (ICC) for the VAS score reported in previous studies was 0.88 [25].

The MMST was used to measure the ROM. The MMST is used for the evaluation of the active ROM of the lumbar spine. A line (lower landmark) horizontal to the posterior superior iliac spine (PSIS) was drawn along the midline of the lumbar spine. The examiner marked the second line 15 cm above the lower landmark. Subsequently, the subject was instructed to actively perform anterior flexion with no increase in pain. The distance from the lower to the higher landmarks was then measured [26]. MMST was reported to be 0.78–0.89 on Pearson correlation coefficient for test–retest reliability and 0.72 on ICCs for inter-rater reliability in previous study [27]. A change of more than 1 cm on MMST score should be considered as MCID [28].

The RMDQ was used to measure the changes in the participants’ disability level. It is composed of a series of 24 questions regarding the disability resulting from low back pain [18]. The total score is 24 points, with higher score indicating greater disability. RMDQ was reported to be 0.72 on test–retest reliability [24] and 0.93 on internal consistency reliability in previous study [14]. MCID for RMDQ was 4 points [29].

### 2.8. Data Analysis

The general characteristics of all subjects showed normal distribution. SPSS version 25.0 statistical software (IBM, Chicago, IL, USA) was used in analysis of all statistical values. Results were presented as mean ± standard deviation. The Kolmogorov–Smirnov test was used to identify normality on the general characteristic of the subjects. The paired t-test was used to compare the pre-intervention and post-intervention results within the groups. One-way analysis of variance was used to identify the significant differences between the preintervention and post-intervention values of each group. Bonferroni correction was used for performing multiple comparisons among the groups. The level of statistical significance was set at 0.05.

## 3. Results

A total of 49 subjects participated in this study. The demographic characteristics of all subjects are shown in Table 1. All the general characteristics of the subjects showed normality and homogeneity.

### 3.1. Pain

The VAS score was assessed to evaluate the pain. The change in pain scores, denoted by the differences between the pre-intervention and post-intervention scores, for the SNAGs with LLLT group, SNAGs group, and control group are shown in Table 2. The SNAGs with LLLT group and SNAGs group showed significant differences between the pre-intervention and post-intervention VAS scores (*p* = 0.000). The inter-group differences in the VAS scores were greater for the SNAGs with LLLT and SNAGs groups than for the control group, and the difference was greater for the SNAGs with LLLT group than for the SNAGs group (*p* = 0.001).

### 3.2. Range of Motion

A significant difference was noted in the MMST scores among the SNAGs with LLLT group (1.43 cm), SNAGs group (1.64 cm) and control group (0.09 cm; *p* = 0.000). The inter-group differences in the ROM were greater for the SNAGs with LLLT and SNAGs groups than for the control group (*p* = 0.001). The pre-intervention and post-intervention differences among the three groups are shown in Table 3.

### 3.3. Physical Disability

The RMDQ score for the SNAGs with LLLT group significantly improved from 15.00 to 5.12, and the RMDQ score for the SNAGs group significantly improved from 15.54 to 10.19. The RMDQ score for the control group significantly improved from 16.12 to 14.38 (Table 4). The inter-group differences in the RMDQ score were greater for the SNAGs with LLLT and SNAGs groups than for the control group, and the difference was greater for the SNAGs with LLLT group than for the SNAGs group (*p* = 0.001).

## 4. Discussion

### 4.1. Pain

The study results indicated that, after administration of combined therapy with SNAGs and LLLT, the pain score evaluated using VAS decreased. The previous studies on the pain-reducing effects of SNAGs can be confirmed. The MCID of the VAS was 20 mm [29]. Regarding the mean difference, the treatment effects of the SNAGs with LLLT (3.17 cm) and SNAGs groups (1.86 cm) may both be considered clinically important and statistically significant in terms of pain improvement. However, the control group (0.67 cm) was not clinically important. The effects of SNAGs on VAS score are already well-known [30].

Hidalgo and Pitance reported significant improvement in VAS in resting between SNAGs (median difference, 1.5 cm) and sham SNAGs (median difference, 0 cm) [3]. Mulligan hypothesized that due to the correction of the positional fault, pain, as a result of muscle imbalance or injury, improved [10]. Once the positional fault is corrected, normal pain and muscle tone are achieved [31]. SNAGs are reported to relieve facet joint capsular strain; hence, ROM improvement and pain reduction are achieved [32]. Vicenzino and Hing reported that the immediate pain relief achieved with SNAGs is possibly due to the non-opioid endogenous pain inhibition pathways [33]. Desensitization of the nervous system through habituation can be achieved by applying progressive mobilization. Habituation mechanisms may reduce pain by inhibiting the presynaptic nerve terminal from transmitting noxious impulses [34]. There are some psychological effects of SNAGs. In a meta-analysis, self-efficacy is reported to be closely associated with distress and pain severity [35]. SNAGs may result in a reduction in fear avoidance; hence, functional improvement is achieved through the increase in the activity level. Therefore, a reduction in fear avoidance through the application of SNAGs could be associated with pain in chronic low back pain patients, enhancing self-efficacy.

LLLT induces a decrease in pain; this can also be confirmed in previous studies. Various effects of LLLT, such as modulation of the healing process, reparation of the damaged neural tissues, and nerve blockage are considered the reasons for the improvement of VAS score. Biostimulation effects of LLLT were reported to improve fibroblast function and modify the inflammation, proliferation, and maturation phases of the healing process. Since axons extend to the surface of the skin, the epidermal neural structure, subcutaneous tissues, sympathetic ganglia, and neuromuscular junctions are all directly affected by LLLT [36]. Type A fibers and slow-conducting type C fibers consisting of peripheral nerve endings of nociceptors, located in the epidermis, are rapidly inhibited [37]. It is suggested that laser-induced nerve block can successfully cause long-term nociception alterations [38]. Repeated laser therapy may reduce nociceptive afferent input in the dorsal horn and facilitate the reorganization of synaptic connections in the central nervous system [39]. Hawkins and Abrahamse suggested that action potentials can be inhibited if LLLT is applied with proper parameters; 30% neural blockage for 24 h can be achieved with LLLT treatment of only 10 to 20 min [40].

Although the effect of SNAG treatment alone was insufficient to qualify for MCID (20 mm), it was significantly sufficient to qualify for MCID by Hägg and Fritzell [29]. This suggests that sole Mulligan treatment is efficient in treating chronic low back pain. However, better results may be obtained on VAS if SNAGs are combined with LLLT (*p* < 0.05). According to Kamal, the significant improvement of combined treatment of SNAGs and LLLT compared to other groups (*p* < 0.05) can be attributed to the desensitization, correction of positional fault, and capsular strain release effects of SNAGs, and the synaptic suppression and healing process stimulation effects of LLLT [16]. Although statistical improvement on VAS was observed in the control group, it did not qualify for MCID for chronic low back pain. Considering these points, it can be said that combined treatment of SNAGs with LLLT and electrotherapy is more effective in reducing pain than applying SNAGs or electrotherapy alone.

### 4.2. Range of Motion (ROM)

In this study, the change in ROM was investigated using MMST to confirm the effect of the intervention. All three groups showed significant differences on MMST (*p* < 0.05). The MMST scores of the SNAGs with LLLT and SNAGs groups were statistically different from that of the control group (*p* < 0.05). The MCID of MMST was 10 mm [28]; the treatment effect of SNAGs with LLLT and SNAGs only may be considered both clinically important and statistically significant in ROM improvement. The result of the control group (1.75 cm) was neither clinically important nor statistically significant.

Because of the capsular strain correction of the lumbar facet joint, MMST improved. Stability, pain, and proprioception are affected by the lumbar facet joints [20]. Therefore, applying SNAGs to mobilize the affected facet joints may release capsular strains, resulting in ROM improvement [41]. As a result, both interventions were statistically significant, and lumbar flexion of the SNAGs group (mean flexion from 4.00° to 56.25°) was more effective when compared to Maitland mobilization (mean lumbar flexion from 4.88° to 37.94°).

The result of inefficiency on ROM in this study is possibly due to the significantly low laser dosage [42]. Additionally, it is reported that LLLT possesses a low to zero evidence of effect on ROM in low back pain patients [43]. It seems that biostimulation and the neurophysiological effects of LLLT are not effective in the management of ROM in chronic low back pain patients even if it is combined with SNAGs. The inefficiency of the unstandardized parameters may have also contributed to these results [43].

### 4.3. Physical Disability

In this study, RMDQ was used to confirm changes in physical disability in patients with low back pain. The inter-group differences in the RMDQ were greater for the SNAGs with LLLT and SNAGs groups than for the control group, and the difference was greater for the SNAGs with LLLT group than for the SNAGs group (*p* = 0.001). The mean differences in RMDQ score were 10.23 for SNAGs with LLLT and 5.35 for the SNAGs group. Considering that the MCID of RMDQ is approximately 4–5 points, the treatment effects of both combinatory treatment of SNAGs with LLLT and SNAGs alone may be considered clinically important and statistically significant [44].

The effects of physical disability were significantly better in the SNAGs with LLLT group and SNAGs group. There are several potential reasons for this improvement. First, SNAGs may inhibit nociceptors. It is reported that the improvement of the physical disability may be due to the correction of the positional fault of the lumbar facet joint; thus, normal function is achieved and muscle guarding around the joint is released. Second, the reduction in fear may have psychological effects on SNAGs. Repeated experiences of painful movement without pain or danger may have reduced the intensity of conditioned response through extinction; thus, the disability is reduced [10]. Combinatory treatment of SNAGs and LLLT can lead to tissue relaxation, increased ROM and reduced fear of body movement compared to sole application of SNAGs. Therefore, it is thought that the level of physical disability evaluated by RMDQ showed a more significant difference for SNAGs with LLT.

This study has the following limitations: it had short intervention period and is comprised of a small sample size. Therefore, it is difficult to generalize the findings to all cases of chronic LBP. Secondly, it was difficult to control all the subjects who dropped out during the intervention. An intention to treat analysis was not performed even though some participants had withdrawn. Lastly, only flexion ROM was used to identify the effect of the treatment. In future studies, rotation and extension ROM should also be included.

## 5. Conclusions

This study was conducted to determine the combined treatment effects of Mulligan mobilization and LLLT on pain, ROM, and physical disability in chronic low back pain patients. Based on the result, pain and function improved more in the SNAGs with LLLT group (*p* < 0.05) compared to the other two study groups. Therefore, it is suggested that combined treatment of Mulligan mobilization and LLLT is an effective method to reduce pain and improve ROM and function in chronic low back pain patients.

## Figures and Tables

**Table 1:** General characteristics of the subjects (*N* = 49).

	SNAGs with LLLT (*n* = 17)	SNAGs (*n* = 16)	Control (*n* = 16)	F	*p*/x^2^
Gender (male/female)	8/9	6/10	8/8	0.06	0.943
Age (years)	41.12 ± 16.34	40.69 ± 13.75	38.06 ± 14.63	0.80	0.453
Height (cm)	167.00 ± 8.55	166.63 ± 12.00	168.63 ± 9.55	1.26	0.293
Weight (kg)	65.24 ± 9.10	63.00 ± 13.47	67.00 ± 14.66	1.52	0.229
Body mass index	23.35 ± 2.49	22.49 ± 2.62	23.36 ± 3.66	1.10	0.341

Mean ± SD; SNAGs = sustained natural apophyseal glides; LLLT = low-level laser therapy.

**Table 2:** Differences in pain (*N* = 49).

	SNAGs + LLLT (*n* = 17) A	SNAGs (*n* = 16) B	Control (*n* = 16) C	F (*p*)	Post-Hoc
Pre-intervention	6.22 ± 0.93	6.25 ± 0.93	6.28 ± 1.04		
Post-intervention	3.05 ± 1.65	4.39 ± 1.01	5.61 ± 1.32		
Pre-post	3.17 ± 1.61	1.86 ± 0.84	0.67 ± 1.02	17.98 (0.001)	A|B|C
*t* (*p*)	8.098 (0.000)	8.830 (0.000)	2.647 (0.18)		

Mean ± SD; SNAGs = sustained natural apophyseal glides; LLLT = low-level laser therapy; VAS = visual analogue scale; *t* value = paired *t*-test; F value = one-way analysis of variance.

**Table 3:** Difference on the range of motion (*N* = 49).

	SNAGs + LLLT(*n* = 17) A	SNAGs (*n* = 16) B	Control (*n* = 16) C	F (*p*)	Post-Hoc
Pre-intervention	18.15 ± 0.95	18.56 ± 1.14	18.12 ± 1.07		
Post-intervention	19.59 ± 1.18	20.21 ± 1.32	18.21 ± 1.37		
Pre-post	1.43 ± 0.74	1.64 ± 1.06	0.09 ± 0.97	13.06 (0.001)	A|B|C
*t(p)*	−7.989 (0.000)	−6.202 (0.000)	−0.386 (0.705)		

Mean ± SD; SNAGs = sustained natural apophyseal glides; LLLT = low-level laser therapy; MMST = modified-modified Schober test; *t* value = paired *t*-test; F value = one-way analysis of variance.

**Table 4:** Difference on function (*N* = 49).

	SNAGs + LLLT (*n* = 17) A	SNAGs (*n* = 16) B	Control (*n* = 16) C	F (*p*)	Post-Hoc
Pre-intervention	15.00 ± 2.44	15.54 ± 4.36	16.12 ± 2.89		
Post-intervention	5.12 ± 2.73	10.19 ± 3.54	14.38 ± 3.03		
Pre-post	10.23 ± 2.70	5.35 ± 4.05	1.75 ± 2.81	28.69 (0.001)	A|B|C
*t* (*p*)	15.60 (0.000)	5.29 (0.000)	2.48 (0.025)		

Mean ± SD; SNAGs = sustained natural apophyseal glides; LLLT = low-level laser therapy; RMDQ = Roland Morris disability questionnaire; *t* value = paired *t*-test; F value = one-way analysis of variance.
